# Economic burden of acute otitis media, pneumonia, and invasive pneumococcal disease in children in the United States after the introduction of 13-valent pneumococcal conjugate vaccines during 2014–2018

**DOI:** 10.1186/s12913-023-09244-7

**Published:** 2023-04-25

**Authors:** Tianyan Hu, Yan Song, Nicolae Done, Salini Mohanty, Qing Liu, Eric M. Sarpong, Esteban Lemus-Wirtz, James Signorovitch, Thomas Weiss

**Affiliations:** 1 Merck & Co., Inc, 126 East Lincoln Ave, P.O. Box 2000, Rahway, NJ 07065 USA; 2 Analysis Group, Inc, Boston, MA USA

**Keywords:** *Streptococcus pneumoniae*, Healthcare resource utilization, Cost analysis, Acute otitis media, Pneumonia, Pneumococcal conjugate vaccine, Invasive pneumococcal disease, Insurance claims

## Abstract

**Background:**

*Streptococcus pneumoniae* remains a leading cause of morbidity, mortality, and healthcare resource utilization (HRU) among children. This study quantified HRU and cost of acute otitis media (AOM), pneumonia, and invasive pneumococcal disease (IPD).

**Methods:**

The IBM MarketScan® Commercial Claims and Encounters and Multi-State Medicaid databases from 2014 to 2018 were analyzed. Children with AOM, all-cause pneumonia, or IPD episodes were identified using diagnosis codes in inpatient and outpatient claims. HRU and costs were described for each condition in the commercial and Medicaid-insured populations. National estimates of the number of episodes and total cost ($US 2019 for each condition were extrapolated using data from the US Census Bureau.

**Results:**

Approximately 6.2 and 5.6 million AOM episodes were identified in commercial and Medicaid-insured children, respectively, during the study period. Mean cost per AOM episode was $329 (SD $1505) for commercial and $184 (SD $1524) for Medicaid-insured children. A total of 619,876 and 531,095 all-cause pneumonia cases were identified among commercial and Medicaid-insured children, respectively. Mean cost per all-cause pneumonia episode was $2304 (SD $32,309) in the commercial and $1682 (SD $19,282) in the Medicaid-insured population. A total of 858 and 1130 IPD episodes were identified among commercial and Medicaid-insured children, respectively. Mean cost per IPD episode was $53,213 (SD $159,904) for commercial and $23,482 (SD $86,209) for the Medicaid-insured population. Nationally, there were over 15.8 million cases of AOM annually, with total estimated cost of $4.3 billion, over 1.5 million cases of pneumonia annually, with total cost of $3.6 billion, and about 2200 IPD episodes annually, for a cost of $98 million.

**Conclusions:**

The economic burden of AOM, pneumonia, and IPD among US children remains substantial. IPD and its manifestations were associated with higher HRU and costs per episode, compared to AOM and all-cause pneumonia. However, owing to their higher frequencies, AOM and all-cause pneumonia were the main contributors to the economic burden of pneumococcal disease nationally. Additional interventions, such as the development of pneumococcal conjugate vaccinees with sustained protection of existing vaccine type serotypes as well as broader inclusion of additional serotypes, are necessary to further reduce the burden of disease caused by these manifestations.

**Supplementary Information:**

The online version contains supplementary material available at 10.1186/s12913-023-09244-7.

## Background

*Streptococcus pneumoniae* is a leading cause of morbidity, mortality, and healthcare resource utilization in children worldwide [1]. The clinical spectrum includes localized disease such as acute otitis media (AOM), and more severe infections such as pneumonia and invasive pneumococcal disease (IPD). IPD is defined as isolation of *S. pneumoniae* from a normally sterile site (e.g., blood or cerebrospinal fluid), and has several manifestations, including meningitis, bacteremia, and bacteremic pneumonia. At least 100 serotypes of *S. pneumoniae* have been identified, which are determined by the surface capsular polysaccharide [2].

AOM is a leading cause of acute care visits and antibiotic prescriptions in children, and *S. pneumoniae* accounts for up to 25% of bacterial isolates in middle ear fluid of young children with AOM [3–7]. Moreover, pneumonia and IPD cause considerable pediatric morbidity and mortality, especially in children aged < 5 years of age, and *S. pneumoniae* is the most common typical cause of bacterial community acquired pneumonia in children [8–11]. AOM, pneumonia, and IPD are also associated with high medical expenditures. AOM was estimated to account for $4 billion annually among children in the United States in 2017, and pneumococcal disease was estimated to account for $3.5 billion direct medical costs in 2004 [3, 6].

The first pneumococcal conjugate vaccine (PCV) was licensed in the US and recommended by the Advisory Committee on Immunization Practices in 2000, as part of the pediatric vaccine schedule [12]. It was a 7-valent PCV (PCV7) and included 7 of the most common serotypes to cause pneumococcal disease. Over time, IPD caused by vaccine serotypes declined, and increases in non-vaccine serotypes increased, due to serotype replacement of non-vaccine for vaccine serotypes [13–15]. In 2010, the 13-valent PCV (PCV13), which protects against 6 additional serotypes, replaced PCV7 in the pediatric vaccine schedule.

Several studies based on real-world data, specifically national surveillance data, have reported substantial healthcare resource use (HRU) and healthcare costs associated with pneumococcal disease. However, these studies were subject to limitations such as lack of recent data and of HRU and cost estimates for individual IPD manifestations [3, 16, 17]. Furthermore, two new vaccines were recently approved by the US Food and Drug Administration for the prevention of invasive and non-invasive pneumococcal disease in adults, and their efficacy and safety profiles among children are under evaluation in clinical trials [18–22]. It is therefore important to quantify the economic burden of pediatric AOM, pneumonia, and IPD comprehensively during recent years. The objectives of this study were to evaluate HRU and costs associated with AOM, all-cause pneumonia, and IPD episodes in children < 18 years of age in the US during the period from 2014 to 2018, and to estimate the number of cases and nationally representative health cost estimates for each of these illnesses. The overarching aim is to provide a more reliable value for the most recent estimate of the societal disease burden of pediatric AOM, pneumonia, and IPD.

## Methods

### Data source

This study was conducted using data from the IBM MarketScan® Commercial Claims and Encounters (CCAE; January 1, 2014 to December 31, 2018) and Multi-State Medicaid databases (January 1, 2014 to December 31, 2018). The CCAE database contains information on enrollment eligibility, demographic data, and medical, surgical and prescription drug utilization and expenditure data for approximately 90 million unique individual employees, their spouses, and dependents covered by employer-sponsored private health insurance. The Multi-State Medicaid database includes similar information for nearly 16 million individuals who were enrolled in Medicaid programs in 12 states. Both databases contain information on inpatient, outpatient, and long-term care services.

### Study design and patient population

This was a retrospective observational study of health care utilization and cost associated with AOM, all-cause pneumonia, and IPD episodes using administrative claims data from children < 18 years of age enrolled in commercial or Medicaid plans at any time from January 1, 2014 to December 31, 2018. Children with AOM, all-cause pneumonia, or an IPD episode were identified and defined in each calendar year using inpatient and outpatient claims with International Classification of Diseases 9/10th Revision, Clinical Modification (ICD-9-CM and ICD-10-CM) codes in any position on the claim record. A disease episode was defined as one or more outpatient and/or inpatient claims. For AOM, a gap of at least 14 days with no claims with AOM-related diagnoses was required to define the start of a new episode. For all-cause pneumonia, a gap of at least 90 days with no pneumonia-related diagnoses was required to define the start of a new episode, following prior retrospective studies of administrative records [23–25]. For IPD, a gap of at least 90 days with no IPD-related diagnoses was also required to define the start of a new episode, consistent with prior studies [23–26].

If there was more than one diagnosis code included on the same claim that fulfilled the definitions for multiple diseases or manifestations, the higher-priority condition took precedent according to the following hierarchy, to avoid double-counting of HRU and costs. IPD episodes received the highest priority, with meningitis first, followed by bacteremia, bacteremic pneumonia, and finally other IPD. All-cause pneumonia was prioritized next, and AOM received the lowest priority. Given that IPD incidence is much lower than pneumonia incidence (approximately 100 times lower) and that pneumonia incidence is much lower than AOM incidence (approximately 10 times lower), the hierarchy is not expected to meaningfully impact the incidence rates of these manifestations. For example, even if 20% of IPD episodes overlap with a pneumonia episode, the calculated IR of pneumonia after applying the hierarchy would only be 0.2% lower than the real value if ignoring the overlapping IPD episodes.

IPD was defined as pneumococcal-specific meningitis, bacteremia, bacteremic pneumonia, or other pneumococcal-specific IPDs (e.g., arthritis, peritonitis, pericarditis, endocarditis, and osteomyelitis). All-cause pneumonia was defined as non-invasive pneumonia of any etiology, including bacterial, viral, and of unknown cause. AOM episodes were further categorized as simple or recurrent based on prior history of AOM. Specifically, an AOM episode was categorized as recurrent if a patient had three or more episodes within a six-month period or four or more episodes within a 12-month period, with at least one episode in the preceding six months, based on clinical guidelines [27, 28]. See Supplemental Table A1 for the specific codes used to identify each disease.

### Study outcomes

Outcomes measures included the number of children with AOM, all-cause pneumonia, and IPD (including meningitis, bacteremia, bacteremic pneumonia, and other IPD) by insurance type, as well as mean age, age category, sex, and health plan type; for the commercially insured population, the region and urbanicity was also reported (see Table 1). HRU was also reported for each illness episode, including the proportion of individuals requiring inpatient (IP), emergency room (ER) visits, outpatient (OP) visits, OP antibiotic prescriptions, and surgical procedures (for AOM only). In addition, the mean length of stay (LOS) per IP admission, the mean number of ER visits, mean number of OP visits, mean number of OP antibiotic prescriptions, and mean number of surgical procedures (for AOM) were calculated for each illness episode. If children were admitted from the ER to the hospital, then the associated ER HRU and costs were included with the IP admission HRU and costs. ER visits that were not associated with an IP admission were classified as outpatient ER visits, and antibiotic prescriptions associated with those visits were classified as OP antibiotic prescriptions. Antibiotic prescriptions are generally classified as OP if they are prescribed in the ER without IP admission, or during an OP visit. For some illness episodes that required a long duration of follow-up care, such as pneumococcal meningitis, all healthcare utilization may not have been captured if an individual changed insurance plan during the study period.

**Table 1:** Demographic characteristics for the commercially insured and Medicaid patients with AOM, pneumonia, and IPD, during 2014–2018

	Commercially insured population			Medicaid insured population		
	AOM	All-cause pneumonia	IPD	AOM	All-cause pneumonia	IPD
**Total number of patients**	***N*** **= 4,157,822**	***N*** **= 600,919**	***N*** **= 796**	***N*** **= 3,758,547**	***N*** **= 511,939**	***N*** **= 1058**
**Age, mean (SD)**	4.9 (4.6)	6.4 (4.8)	5.2 (5.4)	4.1 (4.2)	4.7 (4.4)	4.3 (4.8)
< 2 years, %	29.2	15.7	37.1	36.8	29.1	39.3
2–4 years, %	28.2	27.0	19.8	27.7	28.9	24.0
5–17 years, %	42.6	57.2	43.1	35.5	42.0	36.7
**Male, %**	52.1	53.0	54.8	51.7	53.2	58.4
**Region**						
Northeast, %	17.5	22.2	17.1	–	–	–
North Central, %	22.1	20.9	21.2	–	–	–
South, %	44.4	38.3	41.3	–	–	–
West, %	14.8	17.4	19.2	–	–	–
Missing/unknown, %	1.2	1.2	1.1	–	–	–
**Urbanicity**						
Urban, %	83.1	85.8	84.8	–	–	–
Rural, %	11.9	9.8	11.6	–	–	–
Missing, %	4.9	4.4	3.6	–	–	–
**Health plan types**						
HMO/EPO, %	10.2	11.6	9.0	71.5	67.7	63.0
PPO/POS, %	64.5	63.3	65.2	0.1	0.2	0.3
HDHP/CDHP, %	20.5	20.2	19.1	0.0	0.0	0.0
FFS, %	1.3	1.4	1.8	28.3	32.0	36.4
Missing, %	3.5	3.5	4.9	0.1	0.2	0.3

[1] Patients’ month and day of birth was imputed as July 1st for all patients. Age at onset was calculated as the difference between condition start date and imputed birth date. Patients with negative age at onset were included in the analysis

[2] Patients’ demographic characteristics were first determined by each calendar year and then combined throughout the study period, assuming each year has distinct patient population

[3] For each calendar year, patients’ demographic characteristics were determined at the index episode, which was defined as the first disease episode in the given calendar year

[4] Standard deviations for age in each vaccine period were calculated using the pooled standard deviation of the samples in relevant years

*Abbreviations*: *CDHP* Consumer directed health plan, *EPO* Exclusive provider organization, *FFS*: Fee-for-service, *HDHP* High-deductible health plan, *HMO* Health maintenance organization, *IPD* Invasive pneumococcal disease, *PCV* Pneumococcal conjugate vaccine, *POS* Point of service, *PPO* Preferred provider organization, *SD* Standard deviation

Both capitated and non-capitated plans were included in the cost analysis. Non-capitated plans included Point of Service (POS), Exclusive Provider Organization (EPO), Preferred Provider Organization (PPO), High Deductible Health Plan, Fee-for-Service, and Consumer-Directed Health Plans. Capitated plans included Health Maintenance Organizations (HMOs). Healthcare costs of AOM, all-cause pneumonia, and IPD (including meningitis, bacteremia, bacteremic pneumonia, and other IPD) during the disease episode were assessed.

### Statistical analysis

Baseline characteristics (age, sex, region, urbanicity, and health plan types) were described among patients with AOM, all-cause pneumonia, and IPD episodes during the study period, for the commercially insured population. For the Medicaid insured population, age, sex, and health plan types were described. Data on reported geographic region and urbanicity were not available in the Medicaid databases.

HRU and costs were described for AOM, all-cause pneumonia, and IPD episodes among commercially and Medicaid insured populations. The cost per IP admission, ER visit, OP visit, OP pharmacy prescription, and surgical procedure (AOM only) were calculated among patients who received each service, rather than the total number of episodes. For AOM, HRU and cost were described overall, and further stratified between simple and recurrent AOM episodes, separately. For IPD, HRU and cost were described overall, and further stratified for each manifestation separately. Costs were reported as the total paid amounts for adjudicated claims from payers and out-of-pocket payments by patients, including deductibles, co-payments, and co-insurance. Other patient/family costs (such as transportation and lost productivity) were not included. Costs were reported in 2019 USD; costs incurred prior to 2019 were adjusted for inflation using the Consumer Price Index for medical care [29].

Estimates of the nationally representative number of episodes of each condition were based on the standardization of the disease episode incidence rates observed in the MarketScan database using US Census data by each year, as in prior studies [30]. Nationally representative costs per episode were calculated via direct standardization of costs per episode in the MarketScan population by age, sex, and insurance type (commercial versus Medicaid). Nationally representative total cost estimates were calculated as the product of estimated total episodes in the US population and cost per episode in MarketScan database.

Census data for each study year were obtained from the US Census Bureau database [31, 32]. Estimates of the US population by sex, age, and insurance type were calculated for each study year by applying the average proportion of individuals with private and government health insurance for the 0 to17 year age group across all age-sex categories. The incidence rates of each disease in the general US pediatric population were calculated by multiplying the incidence rates for each age-sex-insurance type group in the MarketScan database with the proportion of that group in the general US pediatric population and summing across all groups. Statistical analyses were conducted using SAS version 9.4 (SAS Institute, Inc., Cary, North Carolina) and R statistical software (R Foundation for Statistical Computing, Vienna, Austria).

## Results

### Demographic characteristics

Demographic characteristics for the commercially and Medicaid-insured populations with AOM, all-cause pneumonia, and IPD during the period from 2014 to 2018 are shown in Table 1. Among children with AOM, the mean age was 4.9 years (standard deviation [SD] 4.6) in the commercially insured and 4.1 years (SD 4.2) in the Medicaid insured population. Slightly more than half of children with AOM were male (52.1% in the commercial and 51.7% in the Medicaid population). Among children with all-cause pneumonia, the mean age was 6.4 years (SD 4.8) in the commercially insured and 4.7 years (SD 4.4) in the Medicaid insured population. Slightly more than half of children with all-cause pneumonia were male (53.0% in the commercial and 53.2% in the Medicaid population). Among children with an IPD episode, the mean age was 5.2 years (SD 5.4) in the commercially insured and 4.3 years (SD 4.8) in the Medicaid insured population. More than half of children with an IPD episode were also male (54.8% in the commercial and 58.4% in the Medicaid population). The total size of the pediatric population at risk in the MarketScan commercial population for the study years and the total number of children with commercial insurance in the US based on data from the US Census Bureau is shown in Supplemental Table A2. The number of children at risk and corresponding national estimates for the Medicaid pediatric population are shown in Supplemental Table A3. The rates of risk factors for pneumococcal disease for the commercially and Medicaid insured patients with AOM, all-cause pneumonia, and IPD during the study period are shown in Supplemental Table A4, revealing that risk factors were generally more common among Medicaid-insured patients compared to commercially insured patients.

In the commercially insured population, the majority of children with AOM, all-cause pneumonia, or an IPD episode were insured through a PPO/POS plan (64.5%, 63.3.%, and 65.2, respectively). In contrast, in the Medicaid insured population, the majority of children with one of these illnesses were insured through an HMO/EPO plan (71.5, 67.7, and 63.0%, respectively). In the commercially insured population, the majority of children with AOM, all-cause pneumonia, or an IPD episode resided in an urban area (83.1, 85.8, and 84.8%, respectively).

### Healthcare resource utilization and costs

The HRU and costs associated with AOM in the commercially and Medicaid insured populations from 2014 to 2018 are shown in Table 2. An average of 7.08 million commercially insured children contributed 5.81 million person-years at risk each year. An average of 4.27 million children with Medicaid insurance plans contributed 3.49 million person-years at risk each year. A total of 6,181,608 AOM episodes were identified from the commercially insured population during this five-year period, among which 5,151,249 (83.3%) were simple AOM and 1,030,359 (16.7%) were recurrent AOM. A total of 5,599,089 AOM episodes were identified in the Medicaid insured population, among which 4,646,941 (83.0%) were simple AOM and 952,148 (17.0%) were recurrent AOM.

**Table 2:** HRU and costs associated with AOM (including recurrent AOM and simple AOM) among commercially and Medicaid insured children aged < 18 years (2014–2018)

	Commercially insured population			Medicaid insured population		
	Overall AOM	Simple AOM	Recurrent AOM	Overall AOM	Simple AOM	Recurrent AOM
**Total number of episodes**	***N*** **= 6,181,608**	***N*** **= 5,151,249**	***N*** **= 1,030,359**	***N*** **= 5,599,089**	***N*** **= 4,646,941**	***N*** **= 952,148**
**HRU**						
**IP admission**						
Proportion of episodes with at least 1 IP admission, %	0.03	0.03	0.03	0.06	0.06	0.06
Total LOS per episode^1^, mean (SD)	3.4 (7.8)	3.5 (7.9)	3.2 (7.4)	3.8 (13.1)	3.9 (14.1)	3.1 (5.0)
**ER visit**						
Proportion of episodes with at least 1 ER visit, %	12.8	13.8	7.9	28.2	30.1	18.9
Number of ER visits per episode^1^, mean (SD)	1.0 (0.2)	1.0 (0.2)	1.1 (0.3)	1.0 (0.2)	1.0 (0.2)	1.1 (0.3)
**OP visit**						
Proportion of episodes with at least 1 OP visit, %	88.8	87.7	93.9	75.0	72.9	85.1
Number of OP visits per episode^1^, mean (SD)	1.2 (0.7)	1.1 (0.7)	1.3 (1.0)	1.2 (1.0)	1.1 (1.0)	1.3 (1.0)
**OP antibiotic prescriptions**						
Proportion of episodes with at least 1 prescription, %	72.0	73.7	63.3	82.2	84.2	72.8
Number of antibiotic prescriptions per episode^1^, mean (SD)	1.2 (0.6)	1.1 (0.5)	1.3 (0.7)	1.1 (0.5)	1.1 (0.5)	1.2 (0.5)
**AOM surgical procedures**						
Proportion of episodes with at least 1 procedure, %	3.8	2.1	12.4	3.8	2.2	11.4
Number of surgical procedures per episode^1^, mean (SD)	1.0 (0.2)	1.0 (0.2)	1.0 (0.1)	1.0 (0.2)	1.1 (0.3)	1.0 (0.1)
**Healthcare costs per episode (in 2019 USD)**^**2**^						
Total costs, mean (SD)	$329 ($1505)	$268 ($1416)	$633 ($1859)	$184 ($1524)	$168 ($1641)	$265 ($713)
IP admission costs^1^, mean (SD)	$17,418 ($60,758)	$17,329 ($62,040)	$17,911 ($53,241)	$8235 ($58,223)	$8519 ($63,321)	$6735 ($10,749)
ER visit costs^1^, mean (SD)	$387 ($698)	$384 ($670)	$413 ($902)	$162 ($214)	$161 ($211)	$166 ($234)
OP visit costs^1^, mean (SD)	$189 ($637)	$164 ($539)	$307 ($964)	$121 ($369)	$109 ($332)	$171 ($494)
OP pharmacy costs^1^, mean (SD)	$29 ($65)	$26 ($55)	$43 ($102)	$30 ($65)	$27 ($60)	$40 ($86)
AOM surgical procedure costs^1^, mean (SD)	$2276 ($3100)	$2290 ($3740)	$2265 ($2437)	$468 ($1737)	$483 ($2270)	$453 ($1005)

^1^The HRU costs are the total payments from payers or out-of-pocket payments by patients. The HRU costs per episode for IP admission, ER visit, OP visit, and OP pharmacy were calculated among patients who received the service rather than the total number of episodes. Costs are reported in 2019 USD

^2^Both capitated and non-capitated plans were included in the HRU cost analyses. Non-capitated plans include EPO, PPO, POS, HDHP, and CDHP. Capitated plans include HMO

*Abbreviations*: *AOM* Acute otitis media, *CDHP* Consumer directed health plan, *EPO* Exclusive provider organization, *ER* Emergency room, *FFS* Fee-for-service, *HDHP* High-deductible health plan, *HMO* Health maintenance organization, *HRU* Healthcare resource utilization, *IP* Inpatient, *IPD* Invasive pneumococcal disease, *LOS* Length of stay, *OP* Outpatient, *PCV* Pneumococcal conjugate vaccine, *POS* Point of service, *PPO* Preferred provider organization, *SD* Standard deviation, *USD* US dollars

Among children with AOM, hospitalization occurred very rarely regardless of insurance type (0.03% of episodes in the commercial and 0.06% in the Medicaid insured population), with a mean LOS of 3.4 days (SD 7.8) and 3.8 days (SD 13.1), respectively. ER visits occurred less frequently in the commercially insured vs. Medicaid insured population (12.8% vs. 28.2%) but OP visits were more common in the commercially insured population (88.8% vs. 75.0%). OP antibiotics were prescribed in most children with AOM, but less commonly in commercially insured compared with the Medicaid insured population (72.0% vs. 82.2%). In the commercially insured population, recurrent AOM was associated with a lower proportion of ER visits (7.9% vs. 13.8%), a slightly higher proportion of OP visits (93.9% vs. 87.7%), a slightly lower proportion of OP antibiotics (63.3% vs. 73.7%), and a higher proportion of surgical procedures (12.4% vs. 2.1%), compared with simple AOM. In the Medicaid insured population, there was a similar pattern of lower proportion of ER visits (18.9% vs. 30.1%), slightly higher proportion of OP visits (85.1% vs. 72.9%), lower proportion of OP prescriptions (72.8% vs. 84.2%), and higher proportion of surgical procedures (11.4% vs. 2.2%) in children with recurrent vs. simple AOM.

The mean total cost per episode of AOM was $329 (SD $1505) in the commercially insured and $184 (SD $1524) in the Medicaid insured population, with higher costs for recurrent vs. simple AOM regardless of insurance type (mean cost $633 vs. $268 in the commercially insured, and $265 vs. $168 in the Medicaid insured). The costs for IP admission, ER visits, OP visits, and surgical procedures were all higher for commercially insured, compared with Medicaid insured patients, but regardless of insurance type, OP visits comprised about half of the total costs ($168 [51.0%] for commercially insured and $91 [49.3%] for Medicaid insured population).

The HRU and costs associated with all-cause pneumonia in the commercially and Medicaid insured populations from 2014 to 2018 are shown in Table 3. A total of 619,876 pneumonia episodes were identified in the commercially insured and 531,095 in the Medicaid insured populations during this five-year period. Among children with all-cause pneumonia, hospitalization was uncommon, occurring in 4.9% of the commercially insured and 8.8% of the Medicaid insured population, with a mean LOS of 5.3 days (SD 13.9) and 6.5 days (SD 17.8) respectively. In a similar pattern as that described for AOM, ER visits occurred less frequently, but OP visits were more common in the commercially insured children with all-cause pneumonia, compared with children insured through Medicaid plans, with ER visits in 22.1% vs. 50.3%, and OP visits in 86.8% vs. 65.2%, respectively. Among children who had OP visits, the mean number of visits was about 1.5 per episode, regardless of insurance. About three-quarters of children with all-cause pneumonia received OP antibiotic prescriptions regardless of insurance type (75.5% in the commercial and 77.7% in the Medicaid insured).

**Table 3:** HRU and costs associated with all-cause pneumonia among commercially and Medicaid insured children aged < 18 years (2014–2018)

	Commercially insured population	Medicaid insured population
**Total number of episodes, N**	***N*** **= 619,876**	***N*** **= 531,095**
**IP admission**		
Proportion of episodes with at least 1 IP admission, %	4.9	8.8
Total LOS per episode^1^, mean (SD)	5.3 (13.9)	6.5 (17.8)
**ER visit**		
Proportion of episodes with at least 1 ER visit, %	22.1	50.3
Number of ER visits per episode^1^, mean (SD)	1.2 (.5)	1.1 (.4)
**OP visit**		
Proportion of episodes with at least 1 OP visit, %	86.8	65.2
Number of OP visits per episode^1^, mean (SD)	1.4 (2.9)	1.5 (5.8)
**OP antibiotic prescriptions**		
Proportion of episodes with at least 1 prescription, %	75.5	77.7
Number of antibiotic prescriptions per episode^1^, mean (SD)	1.5 (1.5)	1.6 (1.7)
**Healthcare costs per episode (in 2019 USD)**^**2**^		
Total costs, mean (SD)	$2304 ($32,309)	$1682 ($19,282)
IP admission costs^1^, mean (SD)	$34,653 ($138,889)	$15,041 ($62,759)
ER visit costs^1^, mean (SD)	$1521 ($5097)	$402 ($1146)
OP visit costs^1^, mean (SD)	$257 ($3208)	$193 ($2611)
OP pharmacy costs^1^, mean (SD)	$44 ($1359)	$54 ($370)

^1^The HRU costs are the total payments from payers or out-of-pocket payments by patients. The HRU costs per episode for IP admission, ER visit, OP visit, and OP pharmacy were calculated among patients who received the service rather than the total number of episodes. Costs are reported in 2019 USD

^2^Both capitated and non-capitated plans were included in the HRU cost analyses. Non-capitated plans include EPO, PPO, POS, HDHP, and CDHP. Capitated plans include HMO

*Abbreviations*: *CDHP* Consumer directed health plan, *EPO* Exclusive provider organization, *ER* Emergency roomm, *FFS* Fee-for-service, *HDHP* High-deductible health plan, *HMO* Health maintenance organization, *HRU* Healthcare resource utilization, *IP* Inpatient, *LOS* Length of stay, *OP* Outpatient, *PCV* Pneumococcal conjugate vaccine, *POS* Point of service, *PPO* Preferred provider organization, *SD* Standard deviation, *USD* US dollars

The mean total cost per episode of all-cause pneumonia was $2304 (SD $32,309) in the commercially insured and $1682 (SD $19,282) in the Medicaid insured population. The costs for all visit types were higher in commercially insured, compared with Medicaid insured population, including IP admission ($34,653 vs. $15,041), ER visits ($1521 vs. $402), and OP visits ($257 vs. $193). Though IP admissions occurred in a small minority of children, they were associated with over half of the total costs for all-cause pneumonia, regardless of insurance type, comprising $1698 (73.7%) of the total cost for commercially insured and $1324 (78.7%) for Medicaid insured children. While we did not investigate the prevalence of comorbidities present in hospitalized children, it is possible that other conditions and risk factors were more common in this population.

The HRU and costs associated with IPD and its manifestations in the commercially and Medicaid insured populations are shown in Table 4. A total of 858 IPD episodes were identified in children with commercial insurance plans (188 meningitis, 354 bacteremia, 253 bacteremic pneumonia, and 63 other IPD), and 1130 IPD episodes were identified in children with Medicaid insurance plans (242 meningitis, 508 bacteremia, 334 bacteremic pneumonia, and 46 other IPD). Among the children with an IPD episode identified, 58.2% with commercial insurance and 61.9% with Medicaid insurance had an IP admission in the MarketScan database; because many of these cases required post-index episode follow-up care, it is likely that the initial IP admission may not have been included in the study period. For the IP admissions identified, the mean LOS was 10.6 days (SD 15.5) for the commercially insured and 11.6 (SD 20.3) for the Medicaid insured population. The number of ER visits was similar regardless of insurance (53.1% vs. 58.7%). OP visits occurred in 59.6% of the commercially insured and 56.5% of the Medicaid population, with of mean of 3.4 visits (SD 11.6) and 5.1 visits (SD 52.0), respectively. OP antibiotics were prescribed in a minority of cases regardless of insurance (19.3% vs. 24.2%), presumably because they were prescribed during the IP admissions.

**Table 4:** HRU and costs associated with meningitis, bacteremia, bacteremic pneumonia, and other IPD among commercially and Medicaid insured children aged < 18 years (2014–2018)

	Overall IPD	Meningitis	Bacteremia	Bacteremic pneumonia	Other IPD
**Commercially insured population**					
**Total number of episodes, N**	***N*** **= 858**	***N*** **= 188**	***N*** **= 354**	***N*** **= 253**	***N*** **= 63**
**IP admission**					
Episodes with at least 1 IP admission, n (%)	499 (58.2%)	97 (51.6%)	171 (48.3%)	211 (83.4%)	20 (31.7%)
Total LOS per episode^1^, mean (SD)	10.6 (15.5)	15.2 (18.1)	8.1 (12.2)	11.1 (16.8)	5.0 (3.6)
**ER visit**					
Episodes with at least 1 ER visit, n (%)	456 (53.1%)	93 (49.5%)	145 (41.0%)	199 (78.7%)	19 (30.2%)
Number of ER visits per episode^1^, mean (SD)	1.1 (0.4)	1.1 (0.4)	1.1 (0.5)	1.1 (0.3)	1.1 (0.2)
**OP visit**					
Episodes with at least 1 OP visit, n (%)	511 (59.6%)	151 (80.3%)	244 (68.9%)	68 (26.9%)	48 (76.2%)
Number of OP visits per episode^1^, mean (SD)	3.4 (11.6)	5.6 (15.3)	2.5 (11.2)	1.9 (2.1)	2.9 (5.6)
**OP antibiotic prescriptions**					
Episodes with at least 1 prescription, n (%)	166 (19.3%)	29 (15.4%)	83 (23.4%)	41 (16.2%)	13 (20.6%)
Number of antibiotic prescriptions per episode^1^, mean (SD)	2.0 (2.3)	2.7 (3.7)	2.0 (2.3)	1.6 (0.9)	1.8 (1.7)
**Healthcare costs per episode (in 2019 USD)**					
Total costs, mean (SD)	$53,213 ($159,904)	$57,722 ($128,864)	$33,306 ($107,859)	$87,658 ($236,710)	$13,288 ($24,197)
IP admission costs^1^, mean (SD)	$86,458 ($200,584)	$100,890 ($159,567)	$65,255 ($146,537)	$101,621 ($255,736)	$37,773 ($27,624)
ER visit costs^1^, mean (SD)	$3032 ($7483)	$4329 ($9245)	$2098 ($2857)	$3249 ($9013)	$1547 ($1106)
OP visit costs^1^, mean (SD)	$2177 ($11,937)	$4368 ($17,667)	$1312 ($9792)	$1285 ($4444)	$940 ($2052)
OP pharmacy costs^1^, mean (SD)	$125 ($426)	$114 ($206)	$91 ($205)	$36 ($44)	$552 ($1284)
**Medicaid insured population**					
**Total number of episodes, N**	***N*** **= 1130**	***N*** **= 242**	***N*** **= 508**	***N*** **= 334**	***N*** **= 46**
**IP admission**					
Episodes with at least 1 IP admission, n (%)	700 (61.9%)	130 (53.7%)	283 (55.7%)	265 (79.3%)	22 (47.8%)
Total LOS per episode^1^, mean (SD)	11.6 (20.3)	16.1 (21.7)	12.0 (25.1)	9.4 (12.9)	5.8 (4.2)
**ER visit**					
Episodes with at least 1 ER visit, n (%)	663 (58.7%)	117 (48.3%)	265 (52.2%)	260 (77.8%)	21 (45.7%)
Number of ER visits per episode^1^, mean (SD)	1.1 (0.3)	1.0 (0.2)	1.1 (0.3)	1.0 (0.2)	1.0 (−)^3^
**OP visit**					
Episodes with at least 1 OP visit, n (%)	638 (56.5%)	194 (80.2%)	306 (60.2%)	102 (30.5%)	36 (78.3%)
Number of OP visits per episode^1^, mean (SD)	5.1 (52.0)	12.9 (93.9)	1.7 (2.7)	1.7 (1.4)	1.6 (0.9)
**OP antibiotic prescriptions**					
Episodes with at least 1 prescription, n (%)	273 (24.2%)	50 (20.7%)	144 (28.3%)	64 (19.2%)	15 (32.6%)
Number of antibiotic prescriptions per episode^1^, mean (SD)	2.5 (5.6)	4.8 (12.1)	2.2 (2.9)	1.7 (1.0)	1.5 (0.6)
**Healthcare costs per episode (in 2019 USD)**^**2**^					
Total costs, mean (SD)	$23,482 ($86,209)	$24,457 ($51,711)	$24,328 ($110,237)	$23,355 ($68,668)	$9957 ($15,422)
IP admission costs^1^, mean (SD)	$36,224 ($106,445)	$39,748 ($57,902)	$42,905 ($145,031)	$28,731 ($76,000)	$20,008 ($16,727)
ER visit costs^1^, mean (SD)	$633 ($1662)	$1082 ($2807)	$503 ($1326)	$574 ($1266)	$488 ($466)
OP visit costs^1^, mean (SD)	$1166 ($14,048)	$3153 ($25,386)	$299 ($786)	$332 ($580)	$195 ($303)
OP pharmacy costs^1^, mean (SD)	$111 ($403)	$268 ($864)	$79 ($144)	$69 ($128)	$40 ($46)

^1^The HRU costs are the total payments from payers or out-of-pocket payments by patients. The HRU costs per episode for IP admission, ER visit, OP visit, and OP pharmacy were calculated among patients who received the service rather than the total number of episodes. Costs are reported in 2019 USD

^2^Both capitated and non-capitated plans were included in the HRU cost analyses. Non-capitated plans include EPO, PPO, POS, HDHP, and CDHP. Capitated plans include HMO

^3^Standard deviations with 0 value due to small sample size were not presented

*Abbreviations*: *CDHP* Consumer directed health plan, *EPO* Exclusive provider organization, *ER* Emergency room, *FFS* Fee-for-service, *HDHP* High-deductible health plan, *HMO* Health maintenance organization, *HRU* Healthcare resource utilization, *IP* Inpatient, *IPD* Invasive pneumococcal disease, *LOS* Length of stay, *OP* Outpatient, *PCV* Pneumococcal conjugate vaccine, *POS* Point of service, *PPO* Preferred provider organization, *SD* Standard deviation, *USD* US dollars

The mean total cost per overall IPD episode was $53,213 (SD $159,904) for the commercially insured and $23,482 (SD $86,209) for the Medicaid population. All visit costs per overall IPD episode were higher in the commercially insured, compared with the Medicaid population, including IP admission ($86,458 vs. $36,224), ER visit ($3032 vs. $633), and OP visits ($2177 vs. $1166). IP admissions comprised the majority of total costs for overall IPD episodes regardless of insurance, including $50,319 (94.6%) of total costs for the commercially insured children, and $22,423 (95.5%) of total costs for children with Medicaid insurance plans. It is noteworthy that this is likely an under-estimate for IP admission costs, because the inpatient IP admission may not have been included in the study period for all children with an IPD episode.

In both the commercially insured and the Medicaid insured population, the MarketScan database included the highest proportion of IP admissions for children with bacteremic pneumonia (83.4 and 79.3%) with LOS of 11.1 (SD 16.8) and 9.4 days (SD 12.9), respectively. The total cost per case of bacteremic pneumonia was much higher in the commercially insured, compared with the Medicaid insured population ($87,658 vs. $23,355) due to the much higher cost of hospitalization ($101,621 vs. $28,731 per admission) in those with commercial insurance. IP admissions comprised the majority of total costs for bacteremic pneumonia regardless of insurance, including $84,752 (96.7%) of total costs for the commercially insured children, and $22,784 (97.6%) of total costs for children with Medicaid insurance plans.

The MarketScan database identified 97 (51.6%) IP admissions for the 188 children with pneumococcal meningitis and commercial insurance plans, and 130 (53.7%) for the 242 children with Medicaid insurance plans. It is probable that all children with meningitis had an IP admission, but only the post-index episode follow-up care was included in the database during the study period for some children. The duration of IP admission was slightly shorter for the children with commercial insurance, compared with Medicaid insurance plans, with mean LOS of 15.2 days (SD 18.1) vs. 16.1 days (SD 21.7), but the cost per hospitalization was much higher for the children with commercial insurance ($100,890 vs. $39,748 per admission). IP admissions comprised the majority of total costs for pneumococcal meningitis regardless of insurance, including $52,059 (90.2%) of total costs for the commercially insured children, and $21,345 (87.3%) of total costs for the children with Medicaid insurance plans.

The MarketScan database identified 171 (48.3%) IP admissions for the 354 children with pneumococcal bacteremia and commercial insurance plans, and 283 (55.7%) for the 508 children with Medicaid insurance plans. It is probable that the majority of children with pneumococcal bacteremia had an IP admission, but only the post-index episode follow-up care was included in the database during the study period for some children, as for meningitis. The duration of IP admission was shorter for the children with commercial insurance, compared with Medicaid insurance plans, with mean LOS of 8.1 days (SD 12.2) vs. 12.0 days (SD 25.1), but the cost per hospitalization was much higher for the children with commercial insurance ($65,255 vs. $42,905 per admission). IP admissions comprised the majority of total costs for pneumococcal bacteremia regardless of insurance, including $31,518 (94.6%) of total costs for the commercially insured children, and $23,898 (98.2%) of total costs for the children with Medicaid insurance plans.

### National Estimates

**Fig. 1:**
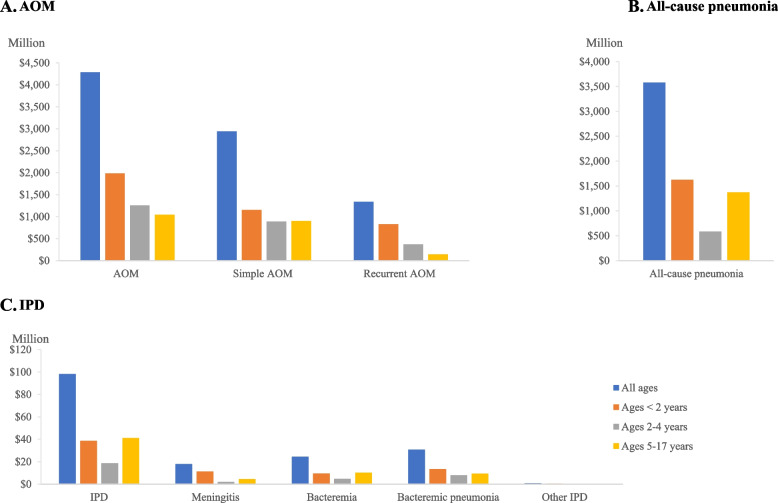
National estimates of mean annual healthcare costs (in millions) of pneumococcal disease in children aged < 18 years in the United States (2014–2018)

The nationally representative healthcare costs of AOM, all-cause pneumonia, and IPD are shown in Fig. 1 and Supplemental Table A5. For AOM, the mean total yearly episodes from 2014 to 2018 were 15,803,584 in US children < 18 years of age, with 6,072,392 episodes among children < 2 years of age, 4,756,047 episodes among children from 2 to 4 years of age, and 4,975,145 episodes among children from 5 to 17 years of age. The mean cost of AOM treatment was $271 per episode at the national level during this time period. Cost per episode was the highest among children of < 2 years of age ($327), followed by those from 2 to 4 years of age ($264) and then by those from 5 to 17 years of age ($210). Recurrent AOM cost $261 per episode more than simple AOM ($487 vs. $226). Mean costs (SD) per AOM episode for each calendar year broken down by type of service are shown in Supplemental Table A6. At the total population level, AOM cost $4.3 billion in each year during the period from 2014 to 2018, with $2.0 billion, $1.3 billion, and $1.0 billion among children < 2 years, 2 to 4 years, and 5 to 17 years of age, respectively. Among the total $4.3 billion, $2.9 billion were associated with simple AOM and $1.3 billion were associated with recurrent AOM.

For all-cause pneumonia, the mean total yearly episodes were 1,506,736 in US children < 18 years of age, with 323,767 episodes among children < 2 years of age, 444,097 episodes among children from 2 to 4 years of age, and 738,872 episodes among children from 5 to 17 years of age. The mean cost of all-cause pneumonia treatment was $2377 per episode at the national level during this time period. Cost per episode was the highest among children < 2 years of age ($5021), followed by those from 5 to 17 years of age ($1858) and 2 to 4 years of age ($1315). Mean costs (SD) per all-cause pneumonia episode for each study calendar year broken down by type of service are shown in Supplemental Table A7. At the total population level, all-cause pneumonia cost $3.6 billion in each year from 2014 to 2018, with $1.6 billion, $0.6 billion, and $1.4 billion from children < 2 years, 2 to 4 years, and 5 to 17 years, respectively.

For overall IPD, the mean total yearly episodes were 2522 in US children < 18 years of age, with 964 episodes among children < 2 years of age, 566 episodes among children from 2 to 4 years of age, and 992 episodes among children from 5 to 17 years of age. The mean cost of treatment for overall IPD was $38,960 per episode at the national level. Cost per episode was the highest among children from 5 to 17 years of age ($41,497), followed by those < 2 years of age ($39,972) and 2 to 4 years of age ($32,790). Mean costs (SD) per IPD episode for each calendar year by type of service and disease manifestation are shown in Supplemental Table A8. At the total population level, IPD cost $98 million in each year from 2014 to 2018, with $39 million, $19 million, and $41 million from children aged < 2 years, 2 to 4 years, and 5 to 17 years of age, respectively.

Among the specific IPD manifestations, bacteremic pneumonia was associated with highest cost per episode ($41,208), followed by meningitis ($32,150), bacteremia ($22,781), and other IPD ($4958). Among the total yearly cost of $98 million due to IPD, $30.7 million were associated with bacteremic pneumonia, followed by $24.4 million associated with bacteremia, $17.8 million associated with meningitis, and 0.8 million associated with other IPD.

## Discussion

This study demonstrates that AOM, pneumonia, and IPD-associated healthcare resource utilization and costs in the US pediatric population remain substantial in recent years. At the national level, there were an estimated 15.8 million episodes of AOM annually among children < 18 years of age from 2014 to 2018, costing an estimated $4.3 billion per year during this period; approximately $2.9 billion were associated with simple AOM and $1.3 billion were with recurrent AOM. Recent incidence estimates suggest that the rates of recurrent AOM episodes and the percentage of recurrent episodes of the total have remained relatively unchanged over the past decade in children of all ages [33]. There were also about 1.5 million cases of all-cause pneumonia annually in children during this time period. Though the case numbers for all-cause pneumonia were much lower than AOM, the overall cost was nearly $3.6 billion per year, due to the much higher cost of treatment per episode, compared with AOM. In contrast, IPD episodes were much rarer, occurring in about 2500 children in the US annually, but due to the high cost of treatment, were still associated with annual costs of $98 million.

In the MarketScan database, about 80% of the AOM episodes were associated with OP visits, and about 15% with ER visits. Outpatient antibiotics were prescribed for the majority of cases. Hospitalizations were very rare, but surgical procedures were performed in about 4% of cases (12% for children with recurrent AOM). Hospitalization was also uncommon for children with all-cause pneumonia, occurring in 5 to 9% of the episodes, and outpatient and/or ER visits occurred for nearly all episodes. In contrast, IP admission was common among children with an IPD episode, occurring in about 60% of cases. Even so, this is likely an underestimate, as just over 50% of children with pneumococcal meningitis identified in the MarketScan database had an associated IP admission, and practice guidelines recommend inpatient admission for all patients with bacterial meningitis [34, 35]. We attribute this finding to the potential inclusion of follow-up care for the index hospitalization in the MarketScan database, with the initial IP admission not occurring during the study period. Due to data limitations, we were not able to identify the index hospitalization in these patients, given changes in insurance coverage over time in both Commercial and Medicaid databases.

HRU associated with AOM, all-cause pneumonia, and IPD episodes estimated in this study are consistent with prior real-world studies. As reported by Tong et al., more than 90% of AOM cases were seen in the OP setting only [36]. Although IP admission was rare for AOM, it was more common in the treatment of pneumonia, as reported by Tong et al., with 7 to 12% among children < 2 years of age, 4% among children from 2 to 4 years of age, and 3% among children from 5 to 17 years of age [10]. Prior studies on HRU associated with IPD were generally restricted to the IP setting only. A recent study by Adil et al. reported a mean LOS of 23.0 hospital days due to bacteremic meningitis among the US pediatric population [35]. As reported from the US Healthcare Cost and Utilization Project, streptococcal meningitis is associated with 15.9 days in hospital [37]. No prior studies were found to assess HRU associated other IPD manifestations, such as invasive pneumococcal arthritis, peritonitis, pericarditis, endocarditis, and osteomyelitis**.**

The estimated cost per AOM episode treated from 2014 to 2018 in this analysis is consistent with results from other published studies. Tong et al. reported an average of $344, $338, $248, and $186 per AOM episode among children < 1 year, 1 year, 2 to 4 years, and 5 to 17 years of age, respectively, during the years of 2013 to 2014 [36]. An earlier study reported that the mean cost per AOM episode ranged from $102 to $255, pending on whether the child was treated in a retail clinic or office visit [38]. In the present study, the average cost per AOM episode was $327, $264, and $210 among children aged < 2 years, 2–4 years, and 5–17 years, respectively.

The costs per pneumonia episode estimated in the current study were about twice as high compared to a prior study. Tong et al. reported an average of $2622, $1255, $923, and $910 per pneumonia episode among children < 1 year of age, 1 year, 2 to 4 years, and 5 to 17 years of age, respectively, in 2013 and 2014 [10]. In the present study, cost per pneumonia episode was estimated as $5021, $1315, and $1858 among children < 2 years, 2 to 4 years, and 5 to 17 years of age. One potential reason is the different approaches used to define pneumonia episode. Tong et al. required a medical claim more than 28 days after previous consultation that had the same diagnosis code to define a new pneumonia episode, while in our study, a 90-day window was used.

The national estimates for annual healthcare costs for AOM in this analysis are similar to those from prior studies. Suaya et al. reported costs of $4.4 billion among children < 9 years of age in 2016 and Tong et al. reported costs of $4.3 billion in 2014 [36, 39]. Costs estimated in earlier studies were slightly lower than our estimates; Zhou et al. reported about $1 billion in 2004 USD among children aged < 2 years (corresponding to about $1.5 billion in 2019 USD) vs. $2.0 billion in this study [40].

Tong et al. reported a total cost of $13.4 billion associated with pneumonia among the overall US population, however, costs among the pediatric population were not reported [10]. Zhou et al. reported an estimate of $377 million in 2004 (equivalent to $580 million in 2019 USD) associated with all-cause pneumonia among children aged < 2 years of age, which is much lower than our estimation of $1.6 billion in 2019 USD. One potential reason is that Zhou et al. only analyzed the MarketScan CCAE data and therefore total medical expenditures were estimated among US children with private insurance.

To our knowledge, no prior studies have specifically estimated healthcare costs associated with the treatment of IPD in the US pediatric population. Huang et al. estimated a direct medical cost of $3.5 billion associated with overall pneumococcal disease, including AOM, pneumonia, and IPD, in the general US population. However, the estimate was based on a micro-costing approach, using annual incidence of disease from CDC’s Active Bacterial Core Surveillance system, HRU associated with pneumococcal disease from literature, and costs of medical services obtained from the public domain based on payment from the payers (e.g., the Centers for Medicare & Medicaid Services Physician Fee Schedule) [3].

The treatment costs per episode of AOM, all-cause pneumonia, and IPD were much higher in the commercially insured, compared with the Medicaid insured population ($329 vs. $184 for AOM; $2304 vs. $1682 for all-cause pneumonia; and $53,213 vs. $23,482 for any IPD episode). The costs for all IP admissions, ER visits, and OP visits were always higher for commercially insured children, but the cost of OP antibiotic prescriptions was generally similar, regardless of insurance type. A recently conducted cross-sectional analysis of a propensity score-matched sample of low-income adults enrolled in health insurance plans also found higher costs in the commercially insured patients, compared with those enrolled in Medicaid insurance plans, with overall health care spending more than 80% higher among those with commercial insurance [41]. A study conducted in children with sickle cell disease also found that children enrolled in Medicaid insurance plans had lower expenditures that children enrolled in commercial insurance plans [42]. This may be due to various factors, such as variations in the management of pneumococcal disease and health care practices across providers and health systems contracting with different insurance groups. However, these differences are difficult to characterize, and are not captured in the dataset. Given that rates of comorbidities known to be risk factors for pneumococcal disease are generally higher in Medicaid-insured children than in those with commercial insurance, it is unlikely that comorbidities explain these differences. A plausible reason why shorter LOS does not correspond to cost savings is that higher hospital reimbursements for commercial insurance than for Medicaid by a factor of 2–3 times [43, 44], and even more in certain regions.

It is also important to emphasize that *S. pneumoniae* is associated with greater clinical severity and a higher likelihood of complications in AOM, and studies of antimicrobial susceptibility in middle ear isolates of children with AOM in a primary care setting have demonstrated increasing resistance to penicillin, amoxicillin, fluoroquinolones, third-generation cephalosporins, and carbapenems among nonvaccine serotypes [45, 46]. Nearly 16 million cases of AOM occur among children in the US each year, and the majority of children receive OP antibiotic prescriptions. The introduction of new PCV vaccinations covering a broader range of serotypes could have a major impact on the number of OP antibiotic prescriptions at the national level.

Though this study used recent data from a large US national representative claims database to comprehensively assess the HRU and costs associated with pneumococcal disease among the US pediatric population, it has several limitations. First, as with any large claims database, miscoding of diagnoses may occur, potentially leading to misclassification and measurement error. Second, we included all etiologies of AOM and pneumonia in this analysis, though *S. pneumoniae* represents only one common bacterial etiology for these conditions [4, 7, 8]. The exact proportion that are due to pneumococci is difficult to define because tympanocentesis is rarely conducted in AOM, and in a substantial proportion of pneumonia cases, the etiology is not identified. Prior studies have estimated that about 12% of outpatient AOM among children aged 0–17 years [3] and about 25% of all AOM in children aged 6–36 months were of pneumococcal etiology, though there is substantial uncertainty around these estimates [47]. Estimates of pneumococcal pneumonia are even more uncertain, with prior studies finding that anywhere between 2.3 and 30% of inpatient pneumonia [3, 48, 49] and about 20% of outpatient pneumonia [3] being of pneumococcal etiology among children aged 0–17 years. This suggests that the HRU and cost burden estimated for AOM and pneumonia are likely overestimated. Third, pathogen-specific IPD episodes caused by *S. pneumoniae* were identified using diagnosis codes; however, lab values for pathogen cultures were not available. Therefore, incidence rates of pneumococcal-specific IPD and its manifestations were likely underestimated. Fourth, HRU and costs associated with IPD, particularly meningitis, are likely underestimated, due to the failure to capture all IP admissions, and the HRU and costs of long-term sequelae were not captured as well. In addition, we did not include patient and family costs (transportation, lost productivity) for any condition, therefore underestimating the societal cost of these diseases. Fifth, disease episodes were defined with a disease hierarchy based on clinical severity to avoid double counting; however, HRU and costs might be misassigned for episodes where multiple diseases were present. Sixth, bacteremic pneumonia episodes were found to have substantially higher costs than all-cause pneumonia, potentially due to complications requiring chest tube placement or surgical intervention. However, we did not specifically analyze data on chest tube placement or surgical interventions. Seventh, although the MarketScan database is considered representative of commercial health plans in the US, it is based on non-random sampling. The national estimates of disease incidence rates are thus based on the assumption that the study sample accurately reflects the overall commercially insured and Medicaid pediatric populations, respectively. In addition, the national estimates also assume that there are no uninsured children. Eighth, our analysis did not examine costs specifically for different antibiotics used to treat pneumococcal disease. Given the wide range of providers and facilities reporting data for the databases analyzed in this study, many with separate formularies with different unit costs for each drug, reporting the cost data with this level of granularity is outside the scope of this study. Finally, while the disease manifestations analyzed here also affect the adult population, quantifying their burden in the adult population is outside the scope of this study. The study focused on children because they are disproportionately affected by these conditions and have a universal vaccination recommendation, as compared to adults, in whom vaccination is recommended only for those with older age or with risk factors.

## Conclusions

The economic burden of AOM, pneumonia, and IPD in the US pediatric population remains substantial, even 20 years after the development of PCVs. At the patient level, pneumococcal disease and its manifestations were associated with higher HRU and costs, compared to AOM and all-cause pneumonia, but AOM was associated with the majority of antibiotic prescriptions. At the US pediatric population level, AOM and all-cause pneumonia are the main contributors to the economic burden of pneumococcal disease. Additional interventions, such as the introduction of new PCV vaccinations covering a broader range of serotypes are necessary to decrease the burden of pneumococcal disease, and reduce antibiotic prescriptions. Based on recent estimates, the additional disease burden that could be averted by these new vaccines could reach up to two billion dollars [50].

## Supplementary Information

**Additional file 1: Supplemental Table A1.** Diagnosis and procedure codes used in the study. **Supplemental Table A2.** Size of MarketScan commercially insured children population at risk in person-years and estimates of the total US pediatric population with commercial insurance (2014–2018). **Supplemental Table A3.** Size of MarketScan Medicaid-insured children population at risk in person-years and estimates of the total US pediatric population with Medicaid coverage (2014–2018). **Supplemental Table A4.** Risk factors for pneumococcal disease among AOM, all-cause pneumonia, and IPD patients aged < 18 years in the 6 months prior to AOM episodes, 2014–2018. **Supplemental Table A5.** National estimates of healthcare costs of pneumococcal disease in children aged < 18 years in the United States (2014–2018). **Supplemental Table A6.** Healthcare costs of AOM in commercially insured children aged < 18 years in the United States in 2019 USD, by type (2014–2018). **Supplemental Table A7.** Healthcare costs of all-cause pneumonia in commercially insured children aged < 18 years in the United States in 2019 USD, by type (2014–2018). **Supplemental Table A8.** Healthcare costs of IPD manifestations in commercially insured children aged < 18 years in the US in 2019 USD, by type (2014–2018).

## Data Availability

The data that support the findings of this study are available from the IBM® MarketScan® Research Databases but restrictions apply to the availability of these data, which were used under license for the current study, and so are not publicly available. Data are however available from the authors upon reasonable request from the corresponding author with permission from IBM® Watson Health™.

## Abbreviations

ACIP: Advisory Committee on Immunization Practices
AOM: Acute otitis media
CCAE: Commercial Claims and Encounters
EPO: Exclusive Provider Organization
ER: Emergency room
HMO: Health Maintenance Organization
HRU: healthcare resource utilization
ICD-9-CM and ICD-10-CM: International Classification of Diseases 9/10th Revision, Clinical Modification
IP: Inpatient
IPD: Invasive pneumococcal disease
LOS: Length of stay
OP: Outpatient
PCV: Pneumococcal conjugate vaccine
PCV13: 13-Valent pneumococcal conjugate vaccine
PCV7: 7-Valent pneumococcal conjugate vaccine
POS: POINT of Service
PPO: Preferred Provider Organization
SD: Standard deviation
US: United States
USD: United States dollars
