# Supplementary material for: Economic burden of acute otitis media, pneumonia, and invasive pneumococcal disease in children in the United States after the introduction of 13-valent pneumococcal conjugate vaccines during 2014–2018
Source: BMC Health Serv Res. 2023 Apr 25;23:398. doi: 10.1186/s12913-023-09244-7 (PMC10127426; doi:10.1186/s12913-023-09244-7)
Supplement: Supplementary file 1 — Additional file 1: Supplemental Table A1. Diagnosis and procedure codes used in the study. Supplemental Table A2. Size of MarketScan commercially insured children population at risk in person-years and estimates of the total US pediatric population with commercial insurance (2014–2018). Supplemental Table A3. Size of MarketScan Medicaid-insured children population at risk in person-years and estimates of the total US pediatric population with Medicaid coverage (2014–2018). Supplemental Table A4. Risk factors for pneumococcal disease among AOM, all-cause pneumonia, and IPD patients aged < 18 years in the 6 months prior to AOM episodes, 2014–2018. Supplemental Table A5. National estimates of healthcare costs of pneumococcal disease in children aged < 18 years in the United States (2014–2018). Supplemental Table A6. Healthcare costs of AOM in commercially insured children aged < 18 years in the United States in 2019 USD, by type (2014–2018). Supplemental Table A7. Healthcare costs of all-cause pneumonia in commercially insured children aged < 18 years in the United States in 2019 USD, by type (2014–2018). Supplemental Table A8. Healthcare costs of IPD manifestations in commercially insured children aged < 18 years in the US in 2019 USD, by type (2014–2018). [file 12913_2023_9244_MOESM1_ESM.pdf]

Supplementary material

Supplemental Table A1. Diagnosis and procedure codes used in the study

<table>
<tr><th>Condition</th><th>Categories</th><th colspan="3">ICD-9-CM</th><th colspan="3">ICD-10-CM</th><th>Descriptions</th></tr>
<tr><td rowspan="18">IPD</td><td rowspan="4">Meningitis</td><td colspan="3">320.1</td><td colspan="3">G00.1</td><td>Pneumococcal meningitis</td></tr>
<tr><td colspan="3">320.2+041.2</td><td colspan="3">G00.2+B95.3</td><td>Streptococcal meningitis+ Pneumococcal infection</td></tr>
<tr><td colspan="3">320.9 +041.2</td><td colspan="3">G00.9+B95.3</td><td>Bacterial meningitis, unspecified + Pneumococcal infection</td></tr>
<tr><td colspan="3">322.9 +041.2</td><td colspan="3">G03.9+B95.3</td><td>Meningitis, unspecified + Pneumococcal infection</td></tr>
<tr><td rowspan="4">Bacteremia</td><td colspan="3">038.2</td><td colspan="3">A40.3</td><td>Pneumococcal septicemia</td></tr>
<tr><td colspan="3">038.0+041.2</td><td colspan="3">A40.9+B95.3</td><td>Streptococcal septicemia + Pneumococcal infection</td></tr>
<tr><td colspan="3">038.9+041.2</td><td colspan="3">A41.9+B95.3</td><td>Unspecified septicemia + Pneumococcal infection</td></tr>
<tr><td colspan="3">790.7+041.2</td><td colspan="3">R78.81+B95.3</td><td>Bacteremia + Pneumococcal infection</td></tr>
<tr><td rowspan="9">Bacteremic pneumonia</td><td colspan="3">510.x+041.2</td><td colspan="3">J86.x+B95.3</td><td>Empyema+ Pneumococcal infection</td></tr>
<tr><td colspan="3">513.0+041.2</td><td colspan="3">J85.1+B95.3</td><td>Abscess of lung + Pneumococcal infection</td></tr>
<tr><td>038.2</td><td rowspan="4">and</td><td rowspan="4">One code from all-cause pneumonia (see below)</td><td>A40.3</td><td rowspan="4">and</td><td rowspan="4">One code from all-cause pneumonia (see below)</td><td></td></tr>
<tr><td>038.0+041.2</td><td>A40.9+B95.3</td><td></td></tr>
<tr><td>038.9+041.2</td><td>A41.9+B95.3</td><td></td></tr>
<tr><td>790.7+041.2</td><td>R78.81+B95.3</td><td></td></tr>
<tr><td>038.0</td><td rowspan="3">and</td><td rowspan="3">481</td><td>A40.9</td><td rowspan="3">and</td><td rowspan="3">J13</td><td></td></tr>
<tr><td>038.9</td><td>A41.9</td><td></td></tr>
<tr><td>790.7</td><td>R78.81</td><td></td></tr>
<tr><td>Other IPD</td><td colspan="3">--</td><td colspan="3">M00.1x</td><td>Pneumococcal arthritis</td></tr>
</table>

		567.1	K65.8+B95.3	Pneumococcal peritonitis
		420.9x+041.2	I30.1+B95.3	Infective pericarditis + Pneumococcal infection
		421.0+041.2	I33.0+B95.3	Acute and subacute bacterial endocarditis + Pneumococcal infection
		421.1/421.9 + 041.2	I33.9 +B95.3	Acute and subacute endocarditis, unspecified+ Pneumococcal infection
		567.23+041.2	K65.2+B95.3	Spontaneous bacterial peritonitis+ Pneumococcal infection
		730.0x, 730.2x +041.2	M86.1x/M86.2x/M86.9+B95.3	Acute or unspecified osteomyelitis+ Pneumococcal infection
		711.0x/711.9x +041.2	M00.0x, M00.2x, M00.8x, M00.9 + B95.3	Pyogenic/unspecified arthritis+ Pneumococcal infection
**Non-invasive pneumonia**	All-cause pneumonia	480.x (480.0-480.3, 480.8, 480.9)	J12.x (J12.0, J12.1, J12.2, J12.3, J12.81, J12.89, J12.9)	Viral pneumonia
		481	J13, J18.1	Pneumococcal pneumonia
		482.x	A48.1, J14, J15.0, J15.1, J15.2x (J15.20, J15.211, J15.212, J15.29), J15.3, J15.4, J15.5, J15.6, J15.8, J15.9	Other bacterial pneumonia
		483.x (483.0, 483.1, 483.8)	J15.7, J16.x (J16.0, J16.8)	Pneumonia due to other specified organism
		484.x (484.1, 484.3, 484.5-484.8)	A22.1, A37.X1, B25.0, B44.0, J17	Pneumonia in infectious diseases classified elsewhere
		485	J18.0	Bronchopneumonia, organism unspecified
		486	J18.2, J18.8, J18.9	Pneumonia, organism unspecified
		487.0	J09.X1, J10.0x (J10.00, J10.01, J10.08), J11.0x (J11.00, J11.08)	Influenza with pneumonia
**OM**	AOM	382.x	H66.xxx - Suppurative and unspecified otitis media<br>H67.x - Otitis media in diseases classified elsewhere	Acute suppurative otitis media, suppurative and unspecified OM
	OME	381.x	H65.xxx - Nonsuppurative otitis media<br>H68.xxx - Eustachian salpingitis and	Nonsuppurative otitis media and Eustachian tube disorders

			obstruction<br>H69.xx - Other and unspecified disorders of Eustachian tube	
		384.0x	H73.0x	Acute myringitis, unspecified
**AOM complications**		384.2	H72	Perforation of tympanic membrane, unspecified ear
		388.6	H92.1x/H92.2x	Otorrhea/otorrhagia
		383.0x	H70.0x	Acute mastoiditis
**AOM procedures**	ICD procedure codes	**ICD-9-CM Procedure Code:** 20.0x (myringotomy)	**ICD-10 Procedure Codes:** 099500Z, 099570Z, 099580Z, 099600Z, 099670Z, 099680Z, 099700Z, 099730Z, 099740Z, 099770Z, 099780Z, 099800Z, 099830Z, 099840Z, 099870Z, 099880Z	Drainage of Right/Left Middle Ear with Drainage Device, Open Approach<br>Drainage of Right/Left Middle Ear with Drainage Device, Via Natural or Artificial Opening<br>Drainage of Right/Left Middle Ear with Drainage Device, Via Natural or Artificial Opening Endoscopic<br>Drainage of Right/Left Tympanic Membrane with Drainage Device, Open Approach<br>Drainage of Right/Left Tympanic Membrane with Drainage Device, Percutaneous Approach<br>Drainage of Right/Left Tympanic Membrane with Drainage Device, Percutaneous Endoscopic Approach<br>Drainage of Right/Left Tympanic Membrane with Drainage Device, Via Natural or Artificial Opening<br>Drainage of Right/Left Tympanic Membrane with Drainage Device, Via Natural or Artificial Opening Endoscopic
	CPT codes	69420		Myringotomy including aspiration and/or Eustachian tube inflation
		69421		Myringotomy including aspiration and/or Eustachian tube inflation requiring general anesthesia
		69433		Tympanostomy requiring insertion of ventilating tube, with local or topical anesthesia
		69436		Tympanostomy requiring insertion of ventilating tube, with general anesthesia

Supplemental Table A2. Size of MarketScan commercially insured children population at risk in person-years and estimates of the total US pediatric population with commercial insurance (2014-2018)

Year	Total population at risk in person years (MarketScan) [1]				Total US commercially insured population estimates[2]			
	**All ages**	**Age <2**	**Age 2-4**	**Age 5-17**	**All ages**	**Age <2**	**Age 2-4**	**Age 5-17**
2014	9,343,998	837,266	1,328,696	7,178,036	42,647,631	4,738,626	7,107,939	30,801,067
2015	5,677,035	510,025	805,890	4,361,120	43,229,136	4,803,237	7,204,856	31,221,042
2016	5,615,207	492,931	800,411	4,321,865	43,725,652	4,858,406	7,287,609	31,579,638
2017	5,253,221	463,883	778,064	4,011,273	44,102,671	4,900,297	7,350,445	31,851,929
2018	5,317,697	487,172	769,136	4,061,389	42,425,300	4,713,922	7,070,883	30,640,494

[1] Patients' month and day of birth was imputed as July 1st for all patients. Age at onset was calculated as the difference between condition start date and imputed birth date.

[2] Data from the US Census Bureau. The number of patients with commercial insurance was estimated using the proportion of patients with employment-based insurance vs. Medicaid for the population under 18 years old.

Supplemental Table A3. Size of MarketScan Medicaid-insured children population at risk in person-years and estimates of the total US pediatric population with Medicaid coverage (2014-2018)

	Total population at risk in person years (MarketScan) [1-2]				Total US Medicaid-covered population estimates[3]			
**Year**	**All ages**	**Age <2**	**Age 2-4**	**Age 5-17**	**All ages**	**Age <2**	**Age 2-4**	**Age 5-17**
2014	5,343,957	712,153	971,404	3,660,400	31,271,369	3,474,597	5,211,895	22,584,877
2015	5,722,235	732,848	1,000,325	3,989,062	30,832,864	3,425,874	5,138,811	22,268,180
2016	5,657,009	684,027	990,031	3,982,951	30,321,348	3,369,039	5,053,558	21,898,751
2017	5,652,255	673,614	1,019,899	3,958,741	29,860,329	3,317,814	4,976,721	21,565,793
2018	4,703,874	577,989	822,895	3,302,990	31,296,069	3,477,341	5,216,011	22,602,716

Supplemental Table A4. Risk factors for pneumococcal disease among AOM, all-cause pneumonia, and IPD patients aged <18 years in the 6 months prior to AOM episodes, 2014-2018

	AOM				All-cause pneumonia				IPD			
	Commercial		Medicaid		Commercial		Medicaid		Commercial		Medicaid	
**Total number of patients, N**	**N = 3,248,907**		**N = 3,161,210**		**N = 495,642**		**N = 430,315**		**N = 560**		**N= 762**	
**Risk factors**												
Chronic heart disease, n (%)	10,583	(0.3%)	15,514	(0.5%)	4,352	(0.9%)	7,622	(1.8%)	50	(8.9%)	92	(12.1%)
Chronic lung disease including asthma, n (%)	84,490	(2.6%)	139,989	(4.4%)	41,103	(8.3%)	61,539	(14.3%)	89	(15.9%)	170	(22.3%)
Diabetes mellitus, n (%)	8,267	(0.3%)	10,402	(0.3%)	2,136	(0.4%)	2,517	(0.6%)	11	(2.0%)	21	(2.8%)
Cerebrospinal fluid leaks, n (%)	51	(0.0%)	64	(0.0%)	11	(0.0%)	24	(0.0%)	5	(0.9%)	6	(0.8%)
Cochlear implant(s), n (%)	221	(0.0%)	256	(0.0%)	25	(0.0%)	30	(0.0%)	0	(0.0%)	1	(0.1%)
Sickle cell disease or other hemoglobinopathies, and anatomic or functional asplenia, n (%)	2,038	(0.1%)	4,780	(0.2%)	1,155	(0.2%)	3,128	(0.7%)	29	(5.2%)	73	(9.6%)
Congenital or acquired immunodeficiency, n (%)	8,370	(0.3%)	9,913	(0.3%)	3,255	(0.7%)	4,231	(1.0%)	97	(17.3%)	149	(19.6%)
HIV infection, n (%)	64	(0.0%)	170	(0.0%)	19	(0.0%)	73	(0.0%)	0	(0.0%)	0	(0.0%)
Chronic renal failure or nephrotic syndrome, n (%)	1,555	(0.0%)	1,474	(0.0%)	527	(0.1%)	622	(0.1%)	11	(2.0%)	15	(2.0%)
Cancer and iatrogenic immunosuppression, including radiation therapy, n (%)	49,028	(1.5%)	158,829	(5.0%)	12,433	(2.5%)	42,360	(9.8%)	82	(14.6%)	162	(21.3%)
Solid organ transplant, n (%)	1,222	(0.0%)	1,456	(0.0%)	750	(0.2%)	1,027	(0.2%)	43	(7.7%)	56	(7.3%)
Alcoholism, n(%)	261	(0.0%)	541	(0.0%)	80	(0.0%)	121	(0.0%)	0	(0.0%)	1	(0.1%)
Chronic liver disease, n (%)	1,233	(0.0%)	2,104	(0.1%)	625	(0.1%)	975	(0.2%)	18	(3.2%)	38	(5.0%)
Multiple myeloma, n (%)	0	(0.0%)	1	(0.0%)	2	(0.0%)	2	(0.0%)	0	(0.0%)	0	(0.0%)
Tobacco use, n(%)	444	(0.0%)	2,720	(0.1%)	105	(0.0%)	547	(0.1%)	0	(0.0%)	2	(0.3%)
Hearing loss, n(%)	2,314	(0.1%)	3,506	(0.1%)	393	(0.1%)	665	(0.2%)	6	(1.1%)	14	(1.8%)
Pre-term birth, n (%)	5,390	(0.2%)	10,682	(0.3%)	1,097	(0.2%)	2,787	(0.6%)	9	(1.6%)	11	(1.4%)

Notes:
[1] Patients' month and day of birth was imputed as July 1st for all patients. Age at onset was calculated as the difference between condition start date and imputed birth date.
[2] Patients' risk factors were firstly determined by each calendar year and then combined for the entire period, assuming each year has distinct patient population.
[3] For each calendar year, the index episode was defined as the first disease episode in the given calendar year.
[4] Patients were required to be continuously enrolled in the health plan for at least 6-months prior to the start of index episode.
[5] For each calendar year, risk factors were determined based on medical claims during the 6-months pre-index period.
Abbreviations: AOM: Acute otitis media; HIV: Human Immunodeficiency Virus; IPD: invasive pneumococcal disease.

Supplemental Table A5. National estimates of healthcare costs of pneumococcal disease in children aged < 18 years in the United States (2014-2018)

	Number of episodes		Healthcare costs (in 2019 USD)		
	Total episodes	Mean yearly episodes	Cost per episode	Total costs (in millions)	Mean yearly costs (in millions)
**AOM**					
**All ages**	89,668,794	15,803,584	$271	$21,418	$4,284
**Ages < 2 years**	30,361,958	6,072,392	$327	$9,928	$1,986
**Ages 2-4 years**	23,780,237	4,756,047	$264	$6,277	$1,255
**Ages 5-17 years**	24,875,727	4,975,145	$210	$5,213	$1,043
**Simple AOM**					
**All ages**	65,265,694	13,053,139	$226	$14,719	$2,944
**Ages < 2 years**	22,466,436	4,493,287	$257	$5,775	$1,155
**Ages 2-4 years**	19,575,330	3,915,066	$226	$4,431	$886
**Ages 5-17 years**	23,223,928	4,644,786	$194	$4,513	$903
**Recurrent AOM**					
**All ages**	13,752,228	2,750,446	$487	$6,700	$1,340
**Ages < 2 years**	7,895,522	1,579,104	$526	$4,152	$830
**Ages 2-4 years**	4,204,907	840,981	$439	$1,847	$369
**Ages 5-17 years**	1,651,799	330,360	$424	$700	$140
**All ages**	7,533,681	1,506,736	$2,377	$17,910	$3,582
**Ages < 2 years**	1,618,835	323,767	$5,021	$8,128	$1,626
**Ages 2-4 years**	2,220,484	444,097	$1,315	$2,919	$584
**Ages 5-17 years**	3,694,362	738,872	$1,858	$6,863	$1,373
**IPD**					
**All ages**	12,610	2,522	$38,960	$491	$98
**Ages < 2 years**	4,822	964	$39,972	$193	$39

**Ages 2-4 years**	2,830	566	$32,790	$93	$19
**Ages 5-17 years**	4,958	992	$41,497	$206	$41
**Meningitis**					
**All ages**	2,770	554	$32,150	$89.1	$17.8
**Ages < 2 years**	1,015	203	$55,478	$56.3	$11.3
**Ages 2-4 years**	523	105	$19,163	$10.0	$2.0
**Ages 5-17 years**	1,233	247	$18,455	$22.8	$4.6
**Bacteremia**					
**All ages**	5,357	1,071	$22,781	$122.0	$24.4
**Ages < 2 years**	2,475	495	$19,171	$47.5	$9.5
**Ages 2-4 years**	1,121	224	$20,698	$23.2	$4.6
**Ages 5-17 years**	1,761	352	$29,183	$51.4	$10.3
**Bacteremic pneumonia**					
**All ages**	3,727	745	$41,208	$153.6	$30.7
**Ages < 2 years**	1,085	217	$62,313	$67.6	$13.5
**Ages 2-4 years**	1,132	226	$35,016	$39.6	$7.9
**Ages 5-17 years**	1,510	302	$30,688	$46.3	$9.3
**Other IPD**					
**All ages**	757	151	$4,958	$3.75	$0.75
**Ages < 2 years**	247	49	$9,345	$2.31	$0.46
**Ages 2-4 years**	55	11	$3,484	$0.19	$0.04
**Ages 5-17 years**	455	91	$2,749	$1.25	$0.25

Abbreviations: AOM: acute otitis media; IPD: invasive pneumococcal disease; USD: US dollars.

Notes:

[1] Costs are reported in 2019 USD.

[2] Incidence rates were adjusted using data from the U.S. Census Bureau, Current Population Survey and Annual Social and Economic Supplements.

[3] The age group specific columns do not add up to the column for all ages due to rounding.

Supplemental Table A6. Healthcare costs of AOM in commercially insured children aged < 18 years in the United States in 2019 USD, by type (2014-2018)

	2014		2015		2016		2017		2018	
**HRU cost, per episode**	**Mean**	**(SD)**	**Mean**	**(SD)**	**Mean**	**(SD)**	**Mean**	**(SD)**	**Mean**	**(SD)**
**Overall AOM**	**N = 1,766,560**		**N = 1,183,769**		**N = 1,136,313**		**N = 1,060,402**		**N = 1,034,564**	
Total costs	$313	($1,709)	$331	($1,257)	$337	($1,388)	$333	($1,329)	$341	($1,679)
IP admission costs	$21,836	($79,911)	$11,295	($10,843)	$15,594	($52,860)	$17,703	($50,781)	$18,619	($72,039)
ER visit costs	$404	($683)	$392	($662)	$386	($705)	$376	($709)	$373	($731)
OP visit costs	$182	($598)	$193	($772)	$192	($609)	$191	($590)	$191	($605)
OP pharmacy costs	$38	($74)	$34	($89)	$27	($55)	$22	($44)	$21	($39)
AOM surgical procedure costs	$2,143	($2,506)	$2,127	($3,886)	$2,318	($3,017)	$2,356	($2,650)	$2,517	($3,394)
**Simple AOM**	**N = 1,488,234**		**N = 974,982**		**N = 940,148**		**N = 882,683**		**N = 865,202**	
Total costs	$262	($1,573)	$268	($1,112)	$271	($1,330)	$268	($1,237)	$276	($1,677)
IP admission costs	$20,299	($76,528)	$11,210	($10,698)	$16,128	($57,158)	$18,561	($54,442)	$19,438	($78,915)
ER visit costs	$400	($611)	$388	($656)	$383	($671)	$374	($706)	$371	($722)
OP visit costs	$161	($475)	$165	($725)	$165	($508)	$164	($463)	$165	($498)
OP pharmacy costs	$34	($69)	$30	($61)	$24	($50)	$20	($41)	$20	($37)
AOM surgical procedure costs	$2,153	($2,823)	$2,149	($4,667)	$2,328	($3,821)	$2,348	($2,854)	$2,565	($4,494)
**Recurrent AOM**	**N = 278,326**		**N = 208,787**		**N = 196,165**		**N = 177,719**		**N = 169,362**	
Total costs	$584	($2,287)	$626	($1,754)	$654	($1,598)	$657	($1,676)	$674	($1,651)
IP admission costs	$30,938	($97,679)	$11,713	($11,611)	$12,521	($8,763)	$12,307	($11,648)	$14,672	($13,872)
ER visit costs	$435	($1,155)	$422	($713)	$415	($941)	$395	($737)	$392	($812)
OP visit costs	$287	($1,006)	$316	($942)	$313	($927)	$313	($971)	$313	($952)
OP pharmacy costs	$56	($95)	$51	($169)	$39	($75)	$32	($55)	$30	($49)
AOM surgical procedure costs	$2,133	($2,170)	$2,110	($3,139)	$2,310	($2,157)	$2,363	($2,473)	$2,477	($2,079)

Abbreviations: AOM: acute otitis media; CDHP: consumer directed health plan; EPO: exclusive provider organization; ER: emergency room; FFS: fee-for-service; HDHP: high-deductible health plan; HMO: health maintenance organization; HRU: healthcare resource utilization; IP: inpatient; LOS: length of stay; OP: outpatient; POS: point of service; PPO: preferred provider organization; SD: standard deviation; USD: US dollars.

Notes:

[1] Patients' month and day of birth was imputed as July 1st for all patients. Age at onset was calculated as the difference between condition start date and imputed birth date. Patients with negative age at onset were included in the analysis.

[2] The HRU costs are the total payments from payers or out-of-pocket payments by patients. The HRU costs per episode for IP admission, ER visit, OP visit, OP pharmacy, and AOM surgical procedure were calculated among patients who received the service rather than the total number of episodes.

[3] Both capitated and non-capitated plans were included in the HRU cost analyses. Non-capitated plans include EPO, PPO, POS, HDHP, and CDHP. Capitated plans include HMO.

[4] AOM surgical procedures were linked to the closest AOM episode for the patient, regardless of whether it happened before or after the episode.

Supplemental Table A7. Healthcare costs of all-cause pneumonia in commercially insured children aged < 18 years in the United States in 2019 USD, by type (2014-2018)

	2014		2015		2016		2017		2018	
**HRU cost, per episode**	**Mean**	**(SD)**	**Mean**	**(SD)**	**Mean**	**(SD)**	**Mean**	**(SD)**	**Mean**	**(SD)**
Total costs	$2,369	($34,021)	$2,295	($33,428)	$2,145	($29,413)	$2,276	($28,649)	$2,429	($34,744)
IP admission costs	$32,162	($138,331)	$35,176	($146,554)	$34,490	($129,489)	$36,627	($125,641)	$37,259	($152,367)
ER visit costs	$1,456	($2,866)	$1,449	($2,501)	$1,541	($9,526)	$1,586	($3,149)	$1,625	($4,292)
OP visit costs	$270	($5,640)	$256	($1,234)	$255	($1,800)	$245	($1,176)	$252	($1,293)
OP pharmacy costs	$52	($264)	$47	($281)	$38	($213)	$48	($3,163)	$36	($698)

Abbreviations: CDHP: consumer directed health plan; EPO: exclusive provider organization; ER: emergency room; FFS: fee-for-service; HDHP: high-deductible health plan; HMO: health maintenance organization; HRU: healthcare resource utilization; IP: inpatient; LOS: length of stay; OP: outpatient; POS: point of service; PPO: preferred provider organization; SD: standard deviation; USD: US dollars.

Notes:

[1] Patients' month and day of birth was imputed as July 1st for all patients. Age at onset was calculated as the difference between condition start date and imputed birth date. Patients with negative age at onset were included in the analysis.

[2] The HRU costs are the total payments from payers or out-of-pocket payments by patients. The HRU costs per episode for IP admission, ER visit, OP visit, OP pharmacy, and AOM surgical procedure were calculated among patients who received the service rather than the total number of episodes.

[3] Both capitated and non-capitated plans were included in the HRU cost analyses. Non-capitated plans include EPO, PPO, POS, HDHP, and CDHP. Capitated plans include HMO.

Supplemental Table A8. Healthcare costs of IPD manifestations in commercially insured children aged < 18 years in the US in 2019 USD, by type (2014-2018)

	2014		2015		2016		2017		2018	
**HRU cost, per episode**	**Mean**	**(SD)**	**Mean**	**(SD)**	**Mean**	**(SD)**	**Mean**	**(SD)**	**Mean**	**(SD)**
**IPD**	**N = 265**		**N = 167**		**N = 151**		**N = 142**		**N = 133**	
Total costs	$57,443	($196,352)	$69,574	($230,603)	$39,405	($76,490)	$43,950	($76,388)	$49,806	($91,899)
IP admission costs	$106,057	($262,304)	$118,323	($294,460)	$55,163	($86,809)	$64,073	($79,092)	$78,168	($106,189)
ER visit costs	$2,586	($3,685)	$3,064	($8,488)	$2,941	($10,027)	$2,977	($4,881)	$3,888	($9,402)
OP visit costs	$1,647	($6,440)	$1,060	($2,374)	$1,930	($9,160)	$5,063	($25,937)	$1,919	($4,936)
OP pharmacy costs	$42	($60)	$340	($868)	$72	($144)	$99	($205)	$61	($141)
**Meningitis**	**N = 52**		**N = 32**		**N = 35**		**N = 33**		**N = 36**	
Total costs	$46,567	($116,705)	$104,540	($235,677)	$40,556	($51,927)	$43,359	($85,304)	$62,072	($87,808)
IP admission costs	$104,291	($164,392)	$190,012	($296,436)	$59,562	($50,611)	$62,466	($74,817)	$99,540	($89,944)
ER visit costs	$2,533	($2,080)	$3,666	($2,582)	$2,479	($1,858)	$5,149	($9,380)	$8,328	($18,397)
OP visit costs	$4,070	($11,627)	$1,982	($3,740)	$4,184	($15,193)	$8,391	($36,330)	$3,466	($5,315)
OP pharmacy costs	$83	($95)	$303	($390)	$71	($45)	$156	($313)	$42	($58)
**Bacteremia**	**N = 125**		**N = 68**		**N = 63**		**N = 48**		**N = 50**	
Total costs	$21,928	($57,859)	$43,395	($185,830)	$35,095	($92,365)	$34,622	($71,628)	$44,509	($108,621)
IP admission costs	$53,413	($82,754)	$87,256	($259,280)	$59,391	($116,590)	$52,767	($81,630)	$79,726	($137,909)
ER visit costs	$2,727	($4,279)	$1,300	($1,210)	$1,581	($1,586)	$2,251	($2,704)	$2,425	($2,427)
OP visit costs	$713	($2,117)	$779	($1,828)	$594	($669)	$5,604	($26,651)	$449	($587)
OP pharmacy costs	$28	($39)	$143	($313)	$90	($178)	$120	($219)	$76	($178)
**Bacteremic pneumonia**	**N = 82**		**N = 54**		**N = 41**		**N = 40**		**N = 36**	
Total costs	$122,648	($324,637)	$94,929	($295,186)	$51,062	($76,686)	$72,325	($85,306)	$55,767	($83,155)
IP admission costs	$143,217	($350,111)	$117,845	($329,759)	$51,903	($76,275)	$77,928	($85,122)	$69,809	($89,457)
ER visit costs	$2,513	($3,765)	$4,067	($12,251)	$4,579	($15,746)	$2,642	($2,476)	$2,686	($3,028)
OP visit costs	$1,197	($3,671)	$833	($1,447)	$322	($402)	$602	($942)	$4,376	($10,832)
OP pharmacy costs	$29	($30)	$63	($64)	$20	($10)	$12	($7)	$52	($74)
**Other IPD**	**N = 6**		**N = 13**		**N = 12**		**N = 21**		**N = 11**	
Total costs	$433	($331)	$15,123	($35,201)	$18,849	($30,008)	$12,154	($19,137)	$14,229	($16,605)
IP admission costs	$0	(--)	$60,520	($52,813)	$34,509	($33,478)	$36,639	($14,442)	$29,402	($8,653)
ER visit costs	$0	(--)	$1,325	($1,177)	$1,280	($1,314)	$1,778	($1,155)	$1,643	($1,066)
OP visit costs	$431	($329)	$458	($734)	$1,805	($3,620)	$1,317	($2,479)	$365	($441)
OP pharmacy costs	$14	(--)	$3,244	($1,562)	$49	($70)	$92	($191)	$10	($2)

Abbreviations: CDHP: consumer directed health plan; EPO: exclusive provider organization; ER: emergency room; FFS: fee-for-service; HDHP: high-deductible health plan; HMO: health maintenance organization; HRU: healthcare resource utilization; IP: inpatient; IPD: invasive pneumococcal disease; LOS: length of stay; OP: outpatient; POS: point of service; PPO: preferred provider organization; SD: standard deviation; USD: US dollars.

Notes:

[1] Patients' month and day of birth was imputed as July 1st for all patients. Age at onset was calculated as the difference between condition start date and imputed birth date. Patients with negative age at onset were included in the analysis.

[2] The HRU costs are the total payments from payers or out-of-pocket payments by patients. The HRU costs per episode for IP admission, ER visit, OP visit, OP pharmacy, and AOM surgical procedure were calculated among patients who received the service rather than the total number of episodes.

[3] Both capitated and non-capitated plans were included in the HRU cost analyses. Non-capitated plans include EPO, PPO, POS, HDHP, and CDHP. Capitated plans include HMO.
